# Effects of phytogenic feed additive on growth performance, gut health, and antioxidant capacity in broiler chickens challenged with pathogenic *Eimeria tenella*

**DOI:** 10.1016/j.psj.2026.106778

**Published:** 2026-03-13

**Authors:** Ju Hee Lee, Da-Hye Kim, Viet Anh Vu, Seong Taek Kim, Gyu Bum Choi, Sun Hoo Moon, Han Wool Kim, Jong Pyo Chae, Ju Kyoung Oh, Kyung-Woo Lee

**Affiliations:** a Department of Animal Science and Technology, Konkuk University, 120 Neungdong-ro, Gwangjin-gu, Seoul 05029, Republic of Korea; b CJ Blossom Park, Suwon, Gyeonggi-do 16495, Republic of Korea

**Keywords:** Phytogenic feed additive, Coccidiosis, *Eimeria tenella*, Broiler chicken

## Abstract

This study aimed to evaluate a dietary phytogenic preparation (**PT**) comprising *Ginkgo biloba* L. leaves and mangosteen extract in broilers challenged with pathogenic *Eimeria tenella*. One-day-old Arbor Acres broilers (*n* = 600) were randomly assigned to a treatment group (six replicate floor pens/treatment, 20 chicks/replicate): non-challenged and control diet-fed negative control group (**NC**); *E. tenella* challenged and control diet-fed positive control group (**PC**); and PC + PT (50, 100, or 200 mg PT/kg of diet). On day 14, all groups except NC were gavaged with 1.0 × 10⁴ oocysts of *E. tenella*. The *Eimeria* challenge impaired (*P* < 0.05) body weight, which was mitigated by dietary PT on days 21 and 28. Furthermore, *E. tenella* challenge increased cecal lesion scores; however, dietary PT preparations reduced (*P* < 0.001) these lesions. Moreover, *Eimeria* challenge impaired ileal morphology; however, dietary PT alleviated these changes. All *E. tenella*-challenged broilers exhibited lower total short-chain fatty acid content in their cecal digesta and partially lowered (*P* = 0.055) ileal digestibility of crude fat being the latter linearly improved by dietary PT preparations (*P* < 0.05). Nitric oxide concentrations in the serum and cecal mucosa were elevated after *Eimeria* challenge; however, they were relieved by dietary PT in a concentration-dependent manner. Serum total cholesterol and high-density lipoprotein cholesterol concentrations in the *Eimeria*-challenged groups were lower than those in the NC group. *Eimeria* challenge also decreased catalase activity in the liver and superoxide dismutase activity in the cecal mucosa, but increased glutathione peroxidase activity in the cecal mucosa compared with that in the NC group. Increasing dietary PT concentrations increased serum (linear effect, *P* = 0.031) and liver (linear effect, *P* = 0.034) glutathione peroxidase levels and superoxide peroxidase activity in the cecal mucosa (quadratic effect, *P* = 0.015). Collectively, dietary PT shows promising potential as a nutritional solution to reduce avian coccidiosis via the modulation of innate and antioxidant markers and the alleviation of *Eimeria*-specific cecal lesions.

## Introduction

Coccidiosis is a major protozoan disease in poultry caused by several species of the genus *Eimeria* that infect specific regions of the gastrointestinal tract with *Eimeria*-specific intestinal lesions (Lee et al., 2022). *Eimeria tenella* is one of the most pathogenic poultry coccidia species that specifically targets the ceca, where it disrupts intestinal function by damaging the mucosal barrier (Bai et al., 2025; Macdonald et al., 2017). The parasite spreads within the host by modulating apoptosis in cecal epithelial cells, thereby contributing to disease pathogenesis (Yan et al., 2015). In addition, *Eimeria* challenge alters the composition and function of the cecal microbiota, impairs nutrient absorption, and consequently impairs the health, productivity, and growth performance of chickens (Choi et al., 2021; Huang et al., 2018; Stanley et al., 2014).

The global economic loss due to avian coccidiosis is estimated to be approximately $13.85 billion, which includes *Eimeria* mortality and the costs of prophylaxis and treatment (Blake et al., 2020). Although coccidiosis in the poultry industry has been managed using synthetic anticoccidials and vaccines, growing concerns regarding the occurrence of drug resistance (Peek and Landman, 2011) and drug residues in meat products (Beyene, 2015; Rana et al., 2019) have led to the development of natural plant-based phytogens (Abd El-Hack et al., 2022; Abdelli et al., 2021; Han et al., 2022; Murugesan et al., 2015).

Among plant-origin bioactive compounds for broiler chickens, ginkgo and mangosteen have shown promising activity in mitigating avian coccidiosis (Niu et al., 2019b; Sriboonyong et al., 2022). *Ginkgo biloba* L. is a traditional herb found in China. The leaves of *G. biloba* contain abundant flavonoids, including quercetin, which modulate antioxidant enzyme activity, thereby enhancing the innate immune defense system (Niu et al., 2017, 2019b; Saeed et al., 2017). Flavonoid compounds present in *G. biloba* leaf extract exert host-protective effects by alleviating oxidative damage and inflammatory responses in intestinal epithelial cells through their antioxidant and anti-inflammatory activities (Niu et al., 2019b; Tan et al., 2020). Dietary quercetin exhibits diverse biological functions, including antioxidant and immune responses in laying hens (Liu et al., 2014; Simitzis et al., 2018) and broiler chickens (Abdel-Latif et al., 2021; Goliomytis et al., 2014; Sun et al., 2022). Correspondingly, *Garcinia mangostana* L. is a tropical fruit indigenous to Southeast Asia that is recognized globally for its distinctive taste and potential health-promoting properties (Obolskiy et al., 2009). Mangosteen products (peel, pericarp, pulp, and seed) are rich in xanthones and other bioactive compounds with potential antioxidant, anti-inflammatory, and antimicrobial activities (Jung et al., 2006; Lim et al., 2013; Rohman et al., 2019; Zarena and Sankar, 2009). Mangosteen extract is particularly rich in xanthone compounds, which have been reported to disrupt cell membranes and impair mitochondrial function, thereby directly inhibiting the invasion and proliferation of protozoa within intestinal epithelial cells (Son et al., 2022; Sriboonyong et al., 2022). Moreover, the potent antioxidant activity of mangosteen peel has been reported in broiler chickens (Kim et al., 2024; Sriboonyong et al., 2022). These phytochemicals exert antioxidant and anti-inflammatory effects through complementary mechanisms, thereby potentially producing synergistic effects (Wagner and Ulrich-Merzenich, 2009). Therefore, the combined use of ginkgo leaf extract, which mediates host-protective effects, and mangosteen extract, which directly suppresses pathogens, may provide complementary benefits by simultaneously mitigating infection-induced intestinal damage while inhibiting protozoan proliferation.

Based on these beneficial antioxidant and immunomodulatory properties of ginkgo and mangosteen (Elmund and Hartrianti, 2020; Niu et al., 2019a), their combined use may serve as an effective nutritional approach to mitigate *E. tenella*-induced intestinal damage. However, studies investigating the efficacy of a blended phytogenic formulation consisting of these plants under field-isolated *E. tenella* infection conditions remain limited. Therefore, this study evaluated whether a phytogenic preparation containing *G. biloba* leaves and *G. mangostana* extract could alleviate *E. tenella*-induced impairments of growth performance, intestinal health, nutrient utilization, and antioxidant capacity in broiler chickens.

## Materials and methods

### Ethical statement

All chickens used in this experiment were cared for according to protocols approved by the Institutional Animal Care and Use Committee at Konkuk University (KU24207).

### Birds, diets, and experimental design

A total of 600 one-day-old broiler chicks (Arbor Acre) were individually weighed upon arrival, placed in 30 floor pens, and subjected to one of the five experimental treatments with six replicates of 20 chicks each. Each pen measured 1 m in width and 2 m in length, and fresh rice husks were used as bedding material. A phytogenic (**PT**) consisting of *G. biloba* leaves and mangosteen was provided by CJ Blossom Park (Suwon, Korea) and *Ginkgo biloba* L. leaves extract and mangosteen (*Garcinia mangostana*) extract were purchased from Shaanxi Huike Botanical Development Co., Ltd. (Shaanxi, China). The inclusion levels were chosen based on the following previous studies. Mangosteen extract has been reported to exhibit anticoccidial efficacy at 250 ppm (Sriboonyong et al., 2022), while quercetin, a major flavonoid component of *Ginkgo biloba* leaves extract, improved intestinal health and reduces oxidative stress at approximately 200 ppm (Abdel-Latif et al., 2021). Based on these findings, we decided to set 200 ppm as the upper limit of inclusion levels to evaluate potential additive or synergistic effects of the combined supplementation.

A basal diet of corn and soybean meal was formulated (Table 1) and used to prepare the experimental diets by mixing the basal diet with PT to obtain final concentrations of 50, 100, or 200 mg PT/kg diet at the expense of celite. At 14 days of age, birds were orally administered saline or *E. tenella* at a dose of 1.0 × 10⁴ oocysts/bird. Broilers treated with saline were considered unchallenged negative control chickens. The room temperature was initially maintained at 31°C and gradually decreased by 2–3°C per week to reach 24°C on day 21. A lighting schedule of 23 h light and 1 h dark (23L:1D) was applied throughout the experimental period. Food and water were provided *ad libitum*.

**Table 1:** Ingredients and chemical composition of the basal diet (%, as-fed basis).

Ingredients	Concentration (g/100 g)
Corn	56.66
Soybean meal, 45.73% CP	29.15
Corn gluten meal 64.78% CP	7.00
Animal fat	2.00
Iodized salt	0.30
Monocalcium phosphate	1.30
_DL_-Methionine, 99%	0.35
_L_-Lysine-HCl, 56%	0.50
_L_-Threonine, 99%	0.10
Ground limestone	1.90
Sodium bicarbonate	0.24
Choline chloride, 50%	0.20
Vitamin premix^1^	0.15
Mineral premix^2^	0.15
Total	100.00
Calculated nutrient composition, %	
AMEn, kcal/kg	3,039
Dry matter	87.9
CP	22.2
Calcium	1.02
Available phosphorus	0.45
Lysine	1.33
Methionine	0.72
Methionine + Cysteine	1.08
Threonine	0.92
Analyzed nutrient composition, %	
Dry matter	88.5
CP	23.2
Crude fat	3.60
Crude fiber	4.03
Crude ash	8.21

Abbreviations: AMEn, nitrogen-corrected apparent metabolizable energy; CP, crude protein.

1 Vitamin premix consisted of the following nutrients per kg of diet: vitamin A, 18,000 IU; vitamin D_3_, 6,000 IU; vitamin E, 120 mg; vitamin K_3_, 6 mg; thiamine, 6 mg; riboflavin, 15 mg; vitamin B_5_, 30 mg; vitamin B_6_, 9 mg; vitamin B_12_, 0.03 mg; niacin, 90 mg; biotin, 0.3 mg; folate, 3 mg.

2 Mineral premix consisted of the following nutrients per kg of diet: Fe, 90 mg; Mn, 180 mg; Zn, 150 mg; I, 1.88 mg; Se, 0.45 mg; Cu, 24 mg; Co, 1.5 mg.

### Growth performance and sample collection

Body weight and feed intake per pen were measured on days 0, 14, 21, 28, and 32. The European production index (**EPI**) was calculated as previously reported (Marcu et al., 2013).

$$\text{EPI}=\frac{Viability\;\left(\%\right)\times ADG\;\left(g/chick/day\right)}{FCR\;(kg\;feed/kg\;gain)\;\times \;10}$$

At 20 days (i.e., six days post *E. tenella* challenge), two chickens per replicate were randomly selected for blood sampling after euthanasia using carbon dioxide. Immediately after blood sampling, the internal organs, including the liver, ileum, and a pair of ceca were sampled and processed. Serum samples were obtained via centrifugation at 200 × *g* for 15 min and stored at −20 °C until analysis. Ceca were opened longitudinally, and cecal digesta was sampled and further processed to measure volatile fatty acids on the day of sampling. The ceca were then washed thrice with ice-cold saline, and the cecal mucosa was obtained by gently scraping the luminal surface of the opened ceca using a sterile scraper. Approximately 1 g of liver and cecal mucosa were mixed with 9 mL of ice-cold phosphate-buffered saline and homogenized using an Ultra-Turrax (Digital Ultra-Turrax T25, IKA, Staufen, Germany). Thereafter, the homogenates were centrifuged at 12,000 × *g* for 10 min at 4°C, and the supernatants were stored at −20°C until analysis. On day 28 d (i.e., 14 days post *E. tenella* challenge), five chickens per replicate were selected and euthanized using carbon dioxide to collect ileal digesta samples for ileal nutrient digestibility measurements. Subsequently, the ileal digesta was pooled per pen and stored at −20 °C until use.

### Mortality rate

Mortality rate (%) was calculated as the number of birds that died during the experimental period divided by the number of birds placed at the start of the trial, multiplied by 100 (Zhang et al., 2018), and was subsequently used to calculate the mortality-corrected feed conversion ratio. All birds that died after *E. tenella* challenge were necropsied to inspect the ceca for *E. tenella*-specific lesions.

### Lesion Score

A pair of ceca sampled on day 20 (i.e., six days post infection) was excised and cut longitudinally, and the intestinal contents were gently removed. Lesion scores were evaluated according to the method reported by Conway and McKenzie (2007), from 0 to 4, with increasing severity. The scores were independently obtained by three observers in a blinded manner.

### Ileal Morphology

On day 20, a 1 cm-long distal ileal segment was sampled 5 cm proximal to the ileocecal valve, fixed in 10% neutral-buffered formalin, and stained with standard hematoxylin and eosin. Villus height, width, and crypt depth were examined using a light microscope (Olympus BX43, Tokyo, Japan) and photographed using a digital camera (eXcope T500, DIXI Science, Daejeon, Republic of Korea). Intact well-oriented villi and crypts (*n* = 10) were counted to determine villus height, villus width, and crypt depth. The ratio of the villus height to crypt depth was then calculated. In addition, villus surface area (*µ*m^2^ × 10^−3^) was calculated using the following formula: villus surface area = villus height × (villus diameter at 1/3 of its height + diameter at 2/3 of its height)/2 (Miles et al., 2006).

### Serum biochemical parameters

Serum samples were analyzed for total cholesterol, triglyceride, high-density lipoprotein (**HDL**) cholesterol, total protein, albumin, globulin, glutamic oxaloacetic transaminase (**GOT**), glutamic pyruvic transaminase (**GPT**), uric acid, and creatinine using an automatic blood chemical analyzer (Film DRI CHEM 7000i; Fuji Film, Tokyo, Japan), following the procedures described by Kim et al. (2020).

### Nitric oxide measurement

Nitric oxide (**NO**) levels in the serum samples and cecal mucosa homogenates were measured using a modified Griess reagent (Sigma-Aldrich, St. Louis, MO, USA). Briefly, 100 μL of serum or cecal mucosal homogenates was mixed with 100 μL of Griess reagent, and the mixture was incubated for 10 min at room temperature. Thereafter, the absorbance was read at a wavelength of 540 nm using a microplate reader (Synergy2, BioTek, Winooski, VT). Nitric oxide concentration was calculated from a standard curve using sodium nitrate, as described previously (Lee et al., 2011).

### Measurement of antioxidant parameters

Serum samples and liver and cecal mucosal homogenates were used to measure antioxidant parameters, including glutathione peroxidase (**GPX**; EnzyChrom glutathione peroxidase assay kit, BioAssay Systems, Hayward, CA, USA), malondialdehyde (**MDA**; OxiSelect thiobarbituric acid reactive substances assay kit, Cell Biolabs, Inc, San Diego, CA, USA), superoxide dismutase (**SOD**; EnzyChrom superoxide dismutase assay kit, BioAssay Systems, Hayward, CA, USA), and catalase (**CAT**; OxiSelect catalase activity assay kit, Cell Biolabs, Inc, San Diego, CA, USA). All assays were performed using commercial kits, according to the manufacturer’s instructions. The results were normalized to the total protein content of each sample. Total protein concentrations in the liver and cecal mucosa were quantified using the Bradford method (Bradford, 1976), with bovine serum albumin as the reference standard.

### Measurement of cecal short chain fatty acid content

On day 20, approximately 1 g of cecal digesta was mixed with 4 mL of cold distilled water. The suspension was then mixed with 0.05 mL of saturated HgCl_2_, 1 mL of 25% H_3_PO_4_, and 0.2 mL of 2% pivalic acid. The mixture was centrifuged at 1000 × *g* for 20 min at 4°C, and the resulting supernatant was collected and stored at −20°C until analysis. The short-chain fatty acid concentration was measured using gas chromatography (6890 Series GC System, HP, Palo Alto, CA, USA).

### Apparent Ileal digestibility of nutrients

From days 21 to 28, the broilers were fed diets supplemented with 2% celite as an indigestible marker to determine the apparent ileal digestibility (**AID**) of nutrients. Feed and ileal digesta were analyzed for acid-insoluble ash (Dourado et al., 2010), dry matter (**DM**; method 930.15; AOAC, 2007), crude protein (**CP**; method 990.03; AOAC, 2007), and crude ash (method 942.05; AOAC, 2007). The AID of the nutrients was calculated using the following equation: AID = 100 − [(acid-insoluble ash in feed, %/acid-insoluble ash in ileal digesta, %) × (nutrient in ileal digesta, %/nutrient in feed, %) × 100]

### Statistical analysis

All data were analyzed using one-way analysis of variance in a completely randomized design with the general linear model procedure in SAS (version 9.4; SAS Institute Inc., Cary, NC, USA). Each pen was considered as an experimental unit. Normality of the data was assessed using the PROC UNIVARIATE procedure. The results are presented as least-squares means with a pooled standard error of the mean. Duncan’s multiple range test was used for post-hoc comparisons among treatment means. In addition, owing to the unequal intervals among dietary phytogenic doses, orthogonal polynomial contrast coefficients were generated using the interactive matrix language procedure. Statistical significance was set at *P* < 0.05, and the trend was set at 0.05 < *P* ≤ 0.10.

## Results

### Growth performance

*Eimeria tenella* challenge reduced (*P* < 0.05) body weight by 13.80% and 15.14% on days 21 and 28 (7 and 14 days post-challenge), respectively, compared with that of the non-challenged control group (Table 2). Moreover, *E. tenella* challenge impaired (*P* < 0.05) growth performance (i.e., body weight, body weight gain, and feed conversion ratio) after 21 and 28 days. Notably, dietary PT did not affect body weight after 14 days; however, PT increased body weight after 21 (linear effect, *P* = 0.060, quadratic effect, *P* = 0.036) and 28 (linear and quadratic effects, *P* < 0.05) days (*P* < 0.05). On day 32, PT induced a quadratic decrease in the body weight of *E. tenella*-infected chicks at the highest production level (Table 2). However, all *E. tenella-*challenged chicks fed diets with or without dietary PT showed impaired body weight gain compared with that in the non-challenged control group. Similarly, feed intake at all ages was not altered (*P* > 0.05) by *E. tenella* challenge or dietary PT supplementation (Table 2). Furthermore, *E. tenella* challenge increased the feed conversion ratio at all ages; however, dietary PT did not mitigate the *E. tenella*-impaired feed conversion ratio. Moreover, *E. tenella* challenge exhibited a 8.5-fold increase in mortality compared with the non-challenged control group (*P* = 0.192) and induced the formation of severe *E. tenella*-specific cecal lesions, including extensive hemorrhage and bloody or caseous cores. *Eimeria tenella* infection increased mortality by 8.5-fold compared to that in the non-challenged control groups. However, increasing dietary PT levels tended to lower (linear effect, *P* = 0.084) the *E. tenella*-mediated increase in mortality by 22.5% to 36.0% compared with that in the challenged control group (Table 2). The European product index remained low (*P* < 0.05) in all *E. tenella*-infected chicks, irrespective of dietary PT concentrations (Table 2).

**Table 2:** Effects of dietary phytogenic on growth performance in broiler chickens challenged with *Eimeria tenella*.

Item	NC^1^	Coccidiosis Challenge^1^				SEM	*P*-value				
		PC	PT 50	PT 100	PT 200		ANOVA		L		Q
BW, g/bird											
Day 0	43.13	-	42.98	43.21	43.16	0.063	0.315	0.432		0.899	
Day 14	413.96	-	414.88	433.68	424.25	11.817	0.780	0.547		0.568	
Day 21	821.56^a^	708.18^b^	721.33^b^	721.70^b^	717.51^b^	26.127	0.039	0.060		0.036	
Day 28	1391.16^a^	1180.53^b^	1177.28^b^	1208.71^b^	1191.37^b^	37.879	0.003	0.019		0.003	
Day 32	1721.40	1597.93	1545.46	1593.78	1605.99	45.014	0.121	0.109		0.042	
BWG, g/bird											
Day 0–14	372.72	-	371.91	392.37	379.07	13.302	0.833	0.697		0.570	
Day 14–21	400.06^a^	300.92^b^	306.45^b^	286.16^b^	295.33^b^	16.298	<0.001	0.722		0.776	
Day 14–28	969.65^a^	773.27^b^	762.39^b^	773.17^b^	769.19^b^	28.513	<0.001	0.992		0.957	
Day 14–32	1299.89^a^	1190.67^ab^	1130.58^b^	1158.24^b^	1183.81^ab^	35.694	0.033	0.871		0.405	
FI, g/bird											
Day 0–14	457.43	-	437.14	471.10	423.50	15.206	0.393	0.326		0.423	
Day 14–21	518.59	472.51	479.02	449.14	470.36	23.533	0.390	0.858		0.679	
Day 14–28	1432.66	1330.05	1297.95	1312.63	1333.13	40.075	0.184	0.858		0.648	
Day 14–32	1947.73	2029.43	1908.11	1898.82	2002.40	68.760	0.701	0.992		0.205	
FCR, g:g											
Day 0–14	1.229	-	1.182	1.205	1.121	0.030	0.239	0.071		0.737	
Day 14–21	1.298^b^	1.568^a^	1.562^a^	1.575^a^	1.605^a^	0.032	<0.001	0.458		0.769	
Day 14–28	1.479^b^	1.720^a^	1.712^a^	1.698^a^	1.750^a^	0.052	0.012	0.726		0.660	
Day 14–32	1.498^b^	1.705^a^	1.690^a^	1.639^ab^	1.698^a^	0.046	0.030	0.912		0.462	
Mortality, %											
Day 14–32	1.80	15.27	9.76	11.75	11.84	0.633	0.192	0.084		0.100	
EPI	393^a^	292^b^	312^b^	313^b^	301^b^	21.981	0.027	0.880		0.525	

Abbreviations: PT, dietary phytogenic containing gingko leaves and mangosteen; BW, body weight; BWG, body weight gain; FI, feed intake; FCR, feed conversion ratio; EPI, European production index; SEM, pooled standard error of the mean; ANOVA, analysis of variance; L, linear; Q, quadratic.

^a,b^Means within the same row that do not share a common superscript are significantly different (*P* < 0.05).

1 NC, unchallenged group; PC, challenged group; PT 50, challenged + 50 mg PT/kg of diet; PT 100, challenged + 100 mg PT/kg of diet; PT 200, challenged + 200 mg PT/kg of diet.

### Lesion score

On day 20 (i.e., six days post *E. tenella* infection), *E. tenella*-specific lesions were highest in the challenged control group (Table 3). Moreover, increasing dietary PT concentrations tended to reduce (quadratic effect, *P* = 0.068) cecal lesions, and chicks fed diets containing 50 mg PT/kg showed numerically fewer cecal lesions than those in the challenged control group. In essence, all *E. tenella*-challenged chicks exhibited ≥2 severe cecal lesions, and *E. tenella*-specific cecal lesions were not detected in the non-challenged control chicks.

**Table 3:** Effects of dietary phytogenic on cecal lesion scores in broiler chickens challenged with *Eimeria tenella*.

Item	NC^1^	Coccidiosis Challenge^1^				SEM	*P*-value		
		PC	PT 50	PT 100	PT 200		ANOVA	L	Q
d 20									
Lesion score	0^c^	2.92^a^	2.38^b^	2.83^a^	2.83^a^	0.090	<0.001	0.514	0.068

Abbreviations: PT, dietary phytogenic containing gingko leaves and mangosteen; SEM, pooled standard error of the mean; ANOVA, analysis of variance; L, linear; Q, quadratic.

^a−c^Means within the same row that do not share a common superscript are significantly different (*P* < 0.05).

1 NC, unchallenged group; PC, challenged group; PT 50, challenged + 50 mg PT/kg of diet; PT 100, challenged + 100 mg PT/kg of diet; PT 200, challenged + 200 mg PT/kg of diet.

### Ileal morphology

The ileal villus height was not affected (*P* > 0.05) by *E. tenella* challenge but linearly decreased (*P* = 0.001) with increasing dietary PT levels (Table 4). Furthermore, *E. tenella* challenge decreased (*P* < 0.05) the ileal villus width; however, increased dietary PT levels linearly widened the villus (*P* = 0.001). All *E. tenella*-challenged chicks, irrespective of dietary PT concentrations, exhibited lower villus surface areas (*P* < 0.05) than those in the non-challenged control groups. Chicks fed diets containing the highest dietary PT levels (200 mg PT/kg diet) exhibited the lowest crypt depth, which caused a linear decrease in crypt depth as dietary PT levels increased (Table 4). Additionally, *E. tenella* challenge tended to lower the villus height-to-crypt depth ratio by an average of 7.75% compared to that in the non-challenged control groups. Increasing the dietary PT levels linearly lowered (*P* = 0.022) the villus height-to-crypt depth ratio in broiler chicks.

**Table 4:** Effect of dietary phytogenic on ileal morphology of broiler chickens challenged with *Eimeria tenella*.

Item	NC^1^	Coccidiosis Challenge^1^				SEM	*P*-value		
		PC	PT 50	PT 100	PT 200		ANOVA	L	Q
Villus height, µm	626.79^a^	651.68^a^	497.82^b^	581.92^ab^	381.50^c^	39.003	<0.001	0.001	0.733
Villus width, µm	176.72^a^	124.10^c^	140.91^bc^	142.67^bc^	156.29^ab^	6.695	<0.001	0.001	0.438
Villus surface area, µm^2^ × 10^−3^	0.111^a^	0.079^b^	0.073^b^	0.079^b^	0.078^b^	0.006	0.001	0.904	0.837
Crypt depth, µm	138.62^a^	158.66^a^	139.29^a^	158.18^a^	107.18^b^	10.429	0.007	0.004	0.223
VH to CD ratio	4.544^a^	4.192^ab^	3.696^bc^	3.714^bc^	3.518^c^	0.176	<0.001	0.022	0.287

Abbreviations: PT, dietary phytogenic containing gingko leaves and mangosteen; SEM, pooled standard error of the mean; ANOVA, analysis of variance; L, linear; Q, quadratic; VH, villus height; CD, crypt depth.

^a−c^Means within the same row that do not share a common superscript are significantly different (*P* < 0.05).

1 NC, unchallenged group; PC, challenged group; PT 50, challenged + 50 mg PT/kg of diet; PT 100, challenged + 100 mg PT/kg of diet; PT 200, challenged + 200 mg PT/kg of diet.

### Cecal short chain fatty acids

After 20 days (i.e., six days post infection), *E. tenella* challenge decreased (*P* < 0.05) the percentages of acetate and short-chain fatty acids but increased (*P* < 0.05) those of propionate, isovalerate, and branched-chain fatty acids compared with those in the non-challenged control groups (Table 5). The relative percentages of isobutyrate, butyrate, and valerate tended (*P* < 0.05) to increase by an average of 1.6- to 2.5-fold in the *E. tenella*-challenged group compared to that in the non-challenged control group. Increasing dietary PT levels altered *E. tenella*-mediated increases in propionate (quadratic effect, *P* = 0.022), isovalerate (linear effect, *P* = 0.016), and valerate (quadratic effect, *P* = 0.075). In addition, increasing dietary PT decreased (quadratic effect, *P* = 0.068) the percentage of acetate in the cecal digesta. However, the relative percentages of short- and branched-chain fatty acids were not altered by the dietary PT.

**Table 5:** Effects of dietary phytogenic on the relative percentages (% of total) of cecal short-chain fatty acids in broiler chickens challenged with *Eimeria tenella*.

Item^2^	NC^1^	Coccidiosis Challenge^1^				SEM	*P*-value		
		PC	PT 50	PT 100	PT 200		ANOVA	L	Q
Acetate	80.18^a^	63.77^b^	58.41^bc^	54.25^c^	58.43^bc^	2.404	<0.001	0.245	0.068
Propionate	5.424^c^	9.951^b^	17.43^a^	14.79^a^	13.95^ab^	1.283	<0.001	0.327	0.022
Isobutyrate	1.159^b^	2.850^ab^	2.875^ab^	3.936^a^	2.942^ab^	0.528	0.042	0.822	0.380
Butyrate	10.69^b^	17.44^ab^	14.05^ab^	18.75^a^	16.19^ab^	2.214	<0.001	0.996	0.927
Isovalerate	1.212^c^	3.076^b^	4.157^ab^	4.464^ab^	5.478^a^	0.496	<0.001	0.016	0.663
Valerate	1.334^b^	2.913^ab^	3.791^a^	5.119^a^	3.015^ab^	0.697	0.017	0.963	0.075
BCFA	3.705^b^	8.839^a^	10.104^a^	12.207^a^	11.435^a^	1.135	<0.001	0.188	0.293
SCFA	96.29^a^	91.16^b^	89.90^b^	87.79^b^	88.56^b^	1.135	<0.001	0.188	0.293

Abbreviations: PT, dietary phytogenic containing gingko leaves and mangosteen; SEM, pooled standard error of the mean; ANOVA, analysis of variance; L, linear; Q, quadratic; BCFA, branched-chain fatty acid; SCFA, short-chain fatty acid.

^a−c^Means within the same row that do not share a common superscript are significantly different (*P* < 0.05).

1 NC, unchallenged group; PC, challenged group; PT 50, challenged + 50 mg PT/kg of diet; PT 100, challenged + 100 mg PT/kg of diet; PT 200, challenged + 200 mg PT/kg of diet.

2 SCFA = acetate + butyrate + propionate; BCFA = isobutyrate + valerate + isovalerate.

### AID of nutrients

The AID of nutrients was determined on day 28 (i.e., 14 days post *E. tenella* infection) to evaluate whether *E. tenella* infection affected nutrient digestibility during the recovery phase after infection (Table 6). The *E. tenella* challenge and dietary PT supplementation did not affect the ileal digestibility of DM, CP, or crude ash after 28 days. The *E. tenella* challenge numerically lowered (*P* = 0.055) ileal crude fat digestibility by an average of 9.40% compared to that in the non-challenged control groups; however, increasing dietary PT levels linearly mitigated (*P* = 0.020) *E. tenella*-induced depression in crude fat digestibility.

**Table 6:** Effects of dietary phytogenic on the apparent ileal digestibility of nutrients in broiler chickens challenged with *Eimeria tenella*.

Item	NC^1^	Coccidiosis Challenge^1^				SEM	*P*-value		
		PC	PT 50	PT 100	PT 200		ANOVA	L	Q
DM, %	93.23	93.83	93.76	93.68	93.57	0.166	0.125	0.247	0.918
CP, %	72.48	74.22	74.54	73.52	73.33	0.964	0.598	0.425	0.994
Crude fat, %	69.01	62.52	62.76	70.02	70.81	2.470	0.055	0.020	0.546
Crude ash, %	27.39	29.58	29.41	29.38	29.20	1.483	0.826	0.870	0.987

Abbreviations: PT, dietary phytogenic containing gingko leaves and mangosteen; SEM, pooled standard error of the mean; ANOVA, analysis of variance; L, linear; Q, quadratic; DM, dry matter; CP, crude protein.

1 NC, unchallenged group; PC, challenged group; PT 50, challenged + 50 mg PT/kg of diet; PT 100, challenged + 100 mg PT/kg of diet; PT 200, challenged + 200 mg PT/kg of diet.

### Nitric oxide concentration

*Eimeria tenella* challenge elevated (*P* < 0.05) NO concentrations in serum samples and cecal mucosa homogenates compared to those in the non-challenged control groups (Table 7). However, increasing dietary PT levels decreased the *E. tenella*-induced increase in NO levels in serum samples (linear effect, *P* = 0.029; quadratic effect, *P* = 0.079) and cecal mucosa homogenates (quadratic effect, *P* = 0.036).

**Table 7:** Effects of dietary phytogenic on nitric oxide levels in the serum and cecal mucosa of broiler chickens challenged with *Eimeria tenella*^1^.

Item	NC^1^	Coccidiosis Challenge^1^				SEM	*P*-value		
		PC	PT 50	PT 100	PT 200		ANOVA	L	Q
Nitric oxide									
Serum, µM	24.73^b^	30.44^a^	26.31^b^	25.24^b^	25.44^b^	1.342	0.031	0.029	0.079
Cecal mucosa, µM/mg protein	39.07^b^	59.60^a^	40.31^b^	47.09^ab^	49.76^ab^	4.557	0.048	0.446	0.036

Abbreviations: PT, dietary phytogenic containing gingko leaves and mangosteen; SEM, pooled standard error of the mean; ANOVA, analysis of variance; L, linear; Q, quadratic.

^a,b^Means within the same row that do not share a common superscript are significantly different (*P* < 0.05).

1 NC, unchallenged group; PC, challenged group; PT 50, challenged + 50 mg PT/kg of diet; PT 100, challenged + 100 mg PT/kg of diet; PT 200, challenged + 200 mg PT/kg of diet.

### Serum biochemical parameters

The total and HDL cholesterol concentrations in serum samples were low (*P* < 0.05) in all *E. tenella*-challenged broilers compared with those in unchallenged broilers (Table 8). Moreover, dietary PT supplementation did not affect total or HDL cholesterol levels in broiler chickens. Furthermore, no substantial *E. tenella*-induced or dietary PT-induced changes were observed in terms of triglycerides, total protein, albumin, globulin, GOT, GPT, or uric acid in serum samples (*P* > 0.05).

**Table 8:** Effects of dietary phytogenic on serum biological parameters in broiler chickens challenged with *Eimeria tenella*.

Item	NC^1^	Coccidiosis Challenge^1^				SEM	*P*-value		
		PC	PT 50	PT 100	PT 200		ANOVA	L	Q
TCHO, mg/dL	108.42^a^	85.92^b^	78.17^b^	85.50^b^	82.75^b^	3.903	<0.001	0.901	0.698
Triglyceride, mg/dL	115.58	86.17	55.50	97.42	101.42	23.130	0.451	0.398	0.713
HDL-CHO, mg/dL	94.58^a^	65.25^b^	60.50^b^	66.25^b^	63.67^b^	4.290	<0.001	0.984	0.954
Total protein, g/dL	2.63	2.50	2.40	2.46	2.48	0.080	0.349	0.917	0.552
Albumin, g/dL	0.95	0.82	0.78	0.82	0.83	0.050	0.166	0.657	0.700
Globulin, g/dL	1.68	1.68	1.63	1.64	1.65	0.044	0.841	0.771	0.508
GOT, U/L	215.00	184.70	169.70	230.20	188.30	20.822	0.268	0.612	0.266
GPT, U/L	3.08	4.08	3.00	3.42	3.33	0.568	0.690	0.537	0.405
Uric acid, mg/dL	10.99	11.21	7.58	10.56	10.38	1.279	0.290	0.865	0.372

Abbreviations: PT, dietary phytogenic containing gingko leaves and mangosteen; SEM, pooled standard error of mean; ANOVA, analysis of variance; L, linear; Q, quadratic; TCHO, total cholesterol; HDL-CHO, high-density lipoprotein cholesterol; GOT, glutamic oxaloacetic transaminase; GPT, glutamic pyruvic transaminase.

^a,b^Means within the same row that do not share a common superscript are significantly different (*P* < 0.05).

1 NC, unchallenged group; PC, challenged group; PT 50, challenged + 50 mg PT/kg of diet; PT 100, challenged + 100 mg PT/kg of diet; PT 200, challenged + 200 mg PT/kg of diet.

### Antioxidant parameters

The effects of *E. tenella* challenge or dietary PT on indicators of antioxidant capacity, including GPX, SOD, CAT, and MDA, were analyzed in the serum, cecal mucosa, and liver homogenates (Table 9). Broilers challenged with *E. tenella* had higher levels of GPX activity but lower levels of SOD activity in cecal mucosa homogenates than those in the non-challenged control groups. Furthermore, dietary PT levels did not affect the *E. tenella*-induced decrease in GPX activity but ameliorated (quadratic effect, *P* = 0.015) the *E. tenella*-induced decrease in SOD activity. In addition, *Eimeria* challenge and dietary PT levels did not influence CAT and MDA levels in cecal mucosa homogenates. Furthermore, GPX activity in the liver homogenates of the *Eimeria*-challenged group was 24.4% lower (*P* = 0.114) than that in the non-challenged control group. However, increasing dietary PT levels (*P* = 0.034) linearly increased GPX activity. In contrast, CAT levels in liver homogenates were lowered (*P* < 0.05) by *Eimeria* challenge but were unaffected by dietary PT levels. Both *Eimeria* challenge and dietary PT levels had no effect on SOD and MDA levels in the liver homogenates. Furthermore, SOD, CAT, and MDA levels in serum samples were not affected (*P* > 0.05) by *E. tenella* challenge or dietary PT supplementation. However, *E. tenella* challenge slightly decreased (*P* > 0.05) GPX activity by 10.95% compared to that in the non-challenged control groups. Suitably, increasing dietary PT levels linearly increased (*P* = 0.031) the *E. tenella*-induced decrease in GPX activity in serum samples.

**Table 9:** Effects of dietary phytogenic on oxidative stress markers in the serum, liver, and cecal mucosa of broiler chickens challenged with *Eimeria tenella*.

Item	NC^1^	Coccidiosis Challenge^1^				SEM	*P*-value		
		PC	PT 50	PT 100	PT 200		ANOVA	L	Q
Serum									
GPX activity, U/L	444.0	395.4	438.8	522.9	494.3	31.105	0.056	0.031	0.108
SOD activity, U/L	2.249	2.251	2.222	2.222	2.227	0.012	0.240	0.331	0.175
CAT activity, U/mL	1.63	1.60	1.61	1.59	1.62	0.022	0.706	0.419	0.657
MDA, µM	12.524	12.287	10.156	10.101	11.123	1.010	0.281	0.934	0.584
Liver									
GPX activity, U/mg of protein	120.98	91.44	113.36	103.33	127.91	10.011	0.114	0.034	0.997
SOD activity, U/mg of protein	0.213	0.236	0.238	0.236	0.233	0.010	0.426	0.793	0.887
CAT activity, U/mg of protein	15.57^a^	7.67^b^	5.77^b^	9.28^b^	9.72^b^	1.583	0.005	0.234	0.822
MDA, nmol/mg of protein	0.825	0.862	0.901	0.714	1.080	0.089	0.147	0.192	0.123
Cecal mucosa									
GPX activity, U/mg of protein	1.95^b^	4.63^a^	3.33^ab^	3.26^ab^	3.59^a^	0.454	0.018	0.259	0.088
SOD activity, U/mg of protein	1.254^a^	0.843^b^	0.987^b^	1.001^b^	0.915^b^	0.059	0.001	0.494	0.015
CAT activity, U/mg of protein	42.55	72.41	50.60	63.27	62.59	11.457	0.126	0.512	0.178
MDA, nmol/mg of protein	7.902	7.755	5.686	6.032	5.547	0.544	0.843	0.405	0.691

Abbreviations: PT, dietary phytogenic containing gingko leaves and mangosteen; SEM, pooled standard error of the mean; ANOVA, analysis of variance; L, linear; Q, quadratic; GPX, glutathione peroxidase; SOD, superoxide dismutase; CAT, catalase; MDA, malondialdehyde.

^a,b^Means within the same row that do not share a common superscript are significantly different (*P* < 0.05).

1 NC, unchallenged group; PC, challenged group; PT 50, challenged + 50 mg PT/kg of diet; PT 100, challenged + 100 mg PT/kg of diet; PT 200, challenged + 200 mg PT/kg of diet.

## Discussion

Coccidiosis remains a major challenge to poultry production owing to its high morbidity and mortality, resistance to conventional anticoccidial drugs, and growing concerns over their residues in meat products that threaten public health. Accordingly, plant-based phytogenic feed additives are promising natural anticoccidials that have been explored in broiler chickens (Abdelli et al., 2021; Han et al., 2022; Saeed and Alkheraije, 2023). Based on previous studies demonstrating the beneficial effects of *G. biloba* leaf and mangosteen extracts in avian coccidiosis (Dong et al., 2020; Sriboonyong et al., 2022), this study hypothesized that a dietary phytogenic blend of *G. biloba* leaves and mangosteen would exert protective effects on broilers challenged with pathogenic *E. tenella*, which is a species frequently detected in the Korean poultry industry (Flores et al., 2022).

The study demonstrated that *E. tenella* infection severely impaired the growth performance of broiler chickens, as evidenced by reduced body weight and body weight gain. These findings are consistent with those of previous studies indicating that *Eimeria* infection reduces growth performance via intestinal damage, reduces nutrient absorption, and increases energy expenditure (Allen and Fetterer, 2002; Teng et al., 2020; Williams, 2005). Although dietary PT did not completely mitigate *E. tenella*-induced growth depression, increasing dietary PT levels either partially or substantially increased the body weight of *E. tenella*-challenged broilers, thereby suggesting that PT may partially support growth performance (Cao et al., 2012; Niu et al., 2017). Nonetheless, the lack of an effect of dietary PT on growth performance (i.e., body weight gain, feed intake, and feed conversion ratio) may be partly related to the pathogenic *Eimeria* strain used in this study. In this study, the pathogenic field isolate of *E. tenella* was used, and it caused severe cecal lesions (≥2) and a high mortality rate. Although dietary PT, especially at 50 mg/kg, tended to decrease cecal lesions (quadratic effect, *P* = 0.068) and mortality (linear effect, *P* = 0.084), the pathogenicity of *E. tenella* may have been so severe that it could not be mitigated by dietary PT.

Cecal lesion scores at six days post infection were the lowest in broilers fed diets containing 50 mg PT/kg of diet showing partial *in vivo* anticoccidial activity. The *in vivo* anticoccidial activities of *G. biloba* leaves or mangosteen extracts (i.e., pericarp, pulp, peel, and seed) have been investigated (Dong et al., 2020; Elmund and Hartrianti, 2020; Niu et al., 2017; Sriboonyong et al., 2022). In addition, the direct killing of *E. tenella* sporozoites *in vitro* by gingko (Son et al., 2023) and mangosteen (Gutierrez-Orozco and Failla, 2013; Son et al., 2022) have been reported, which may explain the PT-mediated decrease in cecal lesions. Intact forms or gut microbiota-altered metabolites of quercetin or alpha mangosteen, which are active components present in gingko and mangosteen, have been detected in distal gut segments (e.g., ceca) upon ingestion (Graf et al., 2006; Gutierrez-Orozco et al., 2015). However, the active components originating from gingko or mangosteen in cecal digesta were not analyzed in this study.

Gut health indicators were evaluated based on ileal morphology and cecal short-chain fatty acid levels. *Eimeria* infection causes severe damage to the gut mucosa and shifts the composition of the gut microbiota, leading to alterations in the composition of short-chain fatty acids. *Eimeria tenella* challenge influenced ileal morphology and the concentrations of short-chain fatty acids in the cecal digesta (Choi et al., 2021). The current study also confirmed that *E. tenella* infection compromised indicators of gut health by lowering ileal morphology (i.e., ileal villus height, width, and surface area) and the relative percentage of total short-chain fatty acids in cecal digesta. However, contrary to expectations, dietary PT did not ameliorate the *E. tenella*-induced decrease in ileal morphology (i.e., villus height and villus height-to-crypt depth ratio) nor the percentages of short-chain fatty acids in the cecal digesta. A clear explanation for the unexpected effects of dietary PT on ileal morphology and cecal short-chain fatty acids is not readily available at this stage. Nonetheless, even with compromised ileal morphology, dietary PT levels did not affect feed intake or feed conversion ratio, which indicates that nutrient digestion and absorption might not be affected. The latter finding might be interpreted as an indirect modulatory effect of dietary PT on ileal morphology by altering the cecal microbial community. It is known that ileal morphology is influenced by the gut microbiota and its metabolites (Bindari and Gerber, 2022). *Eimeria* infection is known to shift the cecal microbiota or short-chain fatty acid concentrations, thereby compromising gut function (Choi et al., 2021; Leung et al., 2019; Liu et al., 2024). In addition, the modulation of the cecal gut microbiota by gingko or mangosteen has been reported in broiler chickens and those challenged with *Eimeria* spp. or *Clostridium perfringens* (Abdel-Latif et al., 2021; Dong et al., 2020; Liu et al., 2023; Sriboonyong et al., 2022; Wang et al., 2018; Zhang et al., 2015). Moreover, branched-chain fatty acids or harmful metabolites, including ammonia, indoles, p-cresols, and phenols, can be produced by cecal microbiota acting on undigested diet-origin or endogenous proteins (Elling-Staats et al., 2022; Liu et al., 2021; Qaisrani et al., 2014). Thus, it is likely that a dietary PT-mediated shift in the community of cecal microbiota could favor short-chain fatty acid (e.g., acetate) reduction but trigger the production of branched-chain fatty acids (i.e., isovalerate and valerate) and harmful byproducts in cecal digesta. These substances could influence the ileal environment via reverse peristalsis of cecal digesta into the ileum. The possibility of reverse peristalsis of cecal digesta into the small intestine has been reported (Elling-Staats et al., 2022; Rinttilä and Apajalahti, 2013; Rodrigues and Choct, 2018). However, these speculations on the triad associations of ileal morphology, cecal microbiota and branched-chain fatty acids by dietary PT need to be carefully interpreted and further studies are warranted to determine the effects of dietary PT on the cecal microbiota of *Eimeria*-challenged chickens.

The AID of DM, CP, and crude ash was not affected by *Eimeria* challenge or dietary PT supplementation. As ileal digesta were sampled 14 days post *E. tenella* infection, broilers may have recovered from the negative effect of *E. tenella* infection on the utilization of nutrients at the ileal level. Indeed, the digestibility of nutrients was impaired seven days post infection; however, this *Eimeria*-mediated effect disappeared 14 or 21 days post infection (Dunaway and Adedokun, 2021; Gautier et al., 2020; Guo et al., 2025). In terms of the ileal digestibility of crude fat, the *Eimeria-*challenged groups exhibited a decreasing tendency of 9.4% compared with that in the non-challenged control group; however, dietary PT supplementation linearly reversed this *E. tenella*-induced decrease in ileal fat digestibility. At this stage, the mechanism(s) of action of dietary PT on increased fat digestibility are not clear, as both *G. biloba* leaves and mangosteen inhibit pancreatic lipase secretions (Bustanji et al., 2011; Chae et al., 2016). In addition, no information is available on whether these plants stimulate the secretion of bile salts, which are essential for efficient lipid digestion and absorption (Lai et al., 2018). However, a few studies have reported that digestibility of nutrients including crude fat could be altered by dietary *G. biloba* leaves in broilers (Ren et al., 2018) and mangosteen peel in ruminants (Phesatcha et al., 2022; Wanapat et al., 2013). Accordingly, further mechanistic investigations are needed to clarify the potential of PT in restoring *E. tenella*-impaired digestibility.

Nitric oxide is a key signaling molecule involved in immune regulation and oxidative stress (Bogdan, 2001). Hence, NO levels were analyzed in the serum and cecal mucosa to determine inflammatory responses under *E. tenella* infection. Under inflammatory conditions, macrophages and endothelial cells upregulate inducible NO synthase (iNOS) and endothelial NO synthase, leading to excessive NO production. Although moderate NO levels are essential for host defense, their overproduction causes them to react with reactive oxygen species to form peroxynitrite and hydroxyl radicals. This results in oxidative stress, tissue damage, and impaired organ function (Allen, 1997; Mishra and Jha, 2019). Furthermore, elevated and uncontrolled NO levels due to *Eimeria* infection have been linked to *Eimeria*-specific gut lesions (Allen, 1997; Castro et al., 2020; Lee et al., 2011). In the current study, NO concentrations in the serum samples and cecal mucosal homogenates were markedly elevated in broilers challenged with *E. tenella*. This suggests exaggerated inflammatory activation and oxidative stress during coccidial infection. The increased NO levels observed in the *E. tenella*-challenged birds may be attributed to the upregulation of iNOS, a key inflammatory mediator activated during *Eimeria* infection (Laurent et al., 2001). However, dietary PT reversed the *E. tenella*-induced increase in NO levels in both serum and cecal mucosa. Previous studies have shown that quercetin (Alharbi et al., 2025) and α-mangostin (Boonprom et al., 2017) reduced the iNOS expression. This implies that dietary PT may attenuate *E. tenella*-mediated inflammation by suppressing iNOS-mediated NO synthesis.

*E. tenella* infection did not affect the levels of GOT and GPT, which are indicators of hepatic function, but lowered total cholesterol and HDL cholesterol levels in serum samples. These findings are similar to those of earlier studies (Cha et al., 2020; Freitas et al., 2008) that reported that *Eimeria* infection lowered serum cholesterol concentrations via the inhibition of *de novo* cholesterol synthesis in the liver of chickens. Furthermore, extensive mucosal injury and compromised intestinal integrity induced by *E. tenella* markedly impair the absorption of nutrients, particularly lipids (Su et al., 2025; Teng et al., 2020; Zhou et al., 2020). However, dietary PT did not affect or reverse the *E. tenella*-mediated cholesterol metabolism. This lack of effect of dietary PT may be attributed to the severe impairment of intestinal lipid absorption caused by *E. tenella* infection, which could overshadow the potential lipid-modulating effects of PT. In addition, the PT levels used in this study may not have been sufficient to counteract the infection-induced alterations in hepatic cholesterol synthesis.

Considering that both *G. biloba* and mangosteen have anti-inflammatory and antioxidant properties (Kim et al., 2024; Lim et al., 2013; Niu et al., 2017, 2019b), antioxidant markers were evaluated in the serum, liver, and cecal mucosa to elucidate the protective mechanisms of dietary PT against coccidial challenge. Accordingly, *E. tenella* challenge lowered the levels of antioxidant indicators in the serum (GPX), liver (CAT), and cecal mucosa (SOD), which was in agreement with earlier findings in *E. tenella*-challenged broilers (Bai et al., 2026). However, *E. tenella* increased GPX activity in the cecal mucosa compared with that in the non-challenged control group. The detrimental effect of *Eimeria* challenge on antioxidant status has been previously reported (Bun et al., 2011; El-Ghareeb et al., 2023; Georgieva et al., 2006). In addition, the increase in GPX activity in cecal mucosa homogenates following *E. tenella* infection can be interpreted as a compensatory response to increased free radical production, as previously reported (Ogwiji et al., 2024). The current study favorably demonstrated an increased antioxidant capacity with dietary PT. Specifically, dietary PT increased GPX activity in serum and liver samples and SOD activity (quadratic increase, *P* = 0.015) in the cecal mucosa. Both GPX and SOD play primary roles in removing peroxides and hydroxy free radicals, thereby protecting cellular membranes from oxidative stress (Surai et al., 2019). This study suggests that dietary PT could enhance antioxidant defense mechanisms owing to the presence of bioactive compounds, such as xanthones (Elmund and Hartrianti, 2020) and flavonoids, in PT (Alharbi et al., 2025; Nijveldt et al., 2001). Furthermore, the *in vivo* antioxidant properties of mangosteen peels (Kim et al., 2024) and *G. biloba* leaves (Niu et al., 2019a) have also been reported in broiler chickens. Finally, PT might regulate the expression of nuclear factor-2 erythroid related factor (Nrf2), which is a key transcription factor controlling the antioxidant responses to oxidative stress (Itoh et al., 2010) as reported elsewhere (Wang et al., 2018; Shehata et al., 2022).

## Conclusions

In conclusion, *E. tenella* infection impairs growth performance, causes severe cecal lesions, and lowers ileal fat digestibility, disrupts gut health indicators (i.e., ileal morphology and cecal volatile fatty acid levels), and reduces antioxidant markers in broilers. Suitably, graded levels of dietary PT mitigate *E. tenella*-mediated decreases in body weight, ileal fat digestibility, and antioxidant status, as well as *E. tenella*-mediated increases in mortality and cecal lesions. Therefore, the study findings indicate that dietary PT can be considered as a nutrition-based strategy to control or mitigate coccidiosis in broiler chickens. However, the present study was conducted under controlled experimental conditions, and thus the efficacy of dietary PT in large-scale commercial farms may vary depending on management practices, environmental factors, and pathogen load. Further studies under commercial production settings are therefore warranted to validate the practical applicability of this phytogenic preparation.

## CRediT authorship contribution statement

**Ju Hee Lee:** Writing – review & editing, Writing – original draft, Formal analysis, Data curation, Conceptualization. **Da-Hye Kim:** Validation, Supervision. **Viet Anh Vu:** Validation. **Seong Taek Kim:** Validation. **Gyu Bum Choi:** Validation. **Sun Hoo Moon:** Validation. **Han Wool Kim:** Resources, Investigation. **Jong Pyo Chae:** Validation. **Ju Kyoung Oh:** Validation. **Kyung-Woo Lee:** Supervision, Project administration, Data curation, Conceptualization.

## Disclosures

The authors declare that they have no known competing financial interests or personal relationships that could have appeared to influence the work reported in this paper.
